# Preoperative Versus Postoperative Chemotherapy With CAPOX Plus Bevacizumab for Resectable Colorectal Liver Metastases: A Randomized Phase II Trial (HiSCO‐01)

**DOI:** 10.1002/ags3.70035

**Published:** 2025-05-09

**Authors:** Yuji Takakura, Katsunori Shinozaki, Satoshi Ikeda, Hiroyuki Egi, Yuzo Hirata, Manabu Shimomura, Takafumi Oshiro, Takao Hinoi, Daisuke Sumitani, Masahiro Nakahara, Masanori Yoshimitsu, Naruhiko Honmyo, Tsuyoshi Kobayashi, Junko Tanaka, Hideki Ohdan, the Hiroshima Surgical Study Group of Clinical Oncology (HiSCO)

**Affiliations:** ^1^ Department of Gastroenterological and Transplant Surgery Hiroshima Prefectural Hospital Hiroshima Japan; ^2^ Division of Clinical Oncology Hiroshima Prefectural Hospital Hiroshima Japan; ^3^ Department of Gastroenterological and Transplant Surgery, Graduate School of Biomedical and Health Sciences Hiroshima University Hiroshima Japan; ^4^ Department of Surgery Chugoku Rosai Hospital Kure Japan; ^5^ Department of Surgery National Hospital Organization Higashihiroshima Medical Centre Higashi‐Hiroshima Japan; ^6^ Department of Surgery JR Hiroshima Hospital Hiroshima Japan; ^7^ Department of Surgery National Hospital Organization Kure Medical Centre Kure Japan; ^8^ Department of Surgery Kure City Medical Association Hospital Kure Japan; ^9^ Department of Surgery Onomichi General Hospital Onomichi Japan; ^10^ Department of Surgery Hiroshima City Asa Hospital Hiroshima Japan; ^11^ Department of Epidemiology, Infectious Disease Control and Prevention, Institute of Biomedical and Health Sciences Hiroshima University Hiroshima Japan

**Keywords:** chemotherapy, colorectal cancer, liver metastases, neoadjuvant, resectable

## Abstract

**Aim:**

NCCN and ESMO guidelines recommend up to 6 months of perioperative oxaliplatin‐based chemotherapy for patients with resectable colorectal liver metastases (CRLM). However, the optimal sequencing and chemotherapy regimen remain unclear.

**Methods:**

We conducted a randomized phase II trial, HiSCO‐01, to compare the outcomes of preoperative (Preop‐group) and postoperative chemotherapy (Postop‐group) with eight cycles of CAPOX plus bevacizumab (CAPOX‐Bev) in patients with resectable CRLM. The primary endpoint was the treatment compliance rate (TCR), defined as the percentage of patients who received at least six cycles of CAPOX‐Bev and underwent R0 resection.

**Results:**

Of the 81 patients enrolled, 76 patients were eligible. TCR was 89.2% in Preop‐group and 71.8% in Postop‐group (*p* = 0.06). The overall incidence of chemotherapy‐related Grade 3 or higher adverse events was similar between the two groups. The postoperative complication rate was comparable except that biliary fistula developed significantly higher in Postop‐group. The 3‐year progression‐free survival and 5‐year overall survival rates were 32.2% and 60.5% in Preop‐group, respectively, and 38.5% and 57.2% in Postop‐group, respectively.

**Conclusion:**

Both preoperative and postoperative CAPOX‐Bev were safely administered, and preoperative chemotherapy showed numerically higher TCR than postoperative chemotherapy. This multimodal approach is highly promising for treating resectable CRLM.

**Trial Registration:**

UMIN Clinical Trial Registry: UMIN000003783

## Graphical abstract

Our aim was to compare the outcomes of preoperative chemotherapy with postoperative chemotherapy in patients with resectable colorectal liver metastases treated with CAPOX‐Bev. We demonstrated that both preoperative and postoperative CAPOX‐Bev were safely administered, and preoperative chemotherapy showed a numerically higher treatment compliance rate than postoperative chemotherapy.

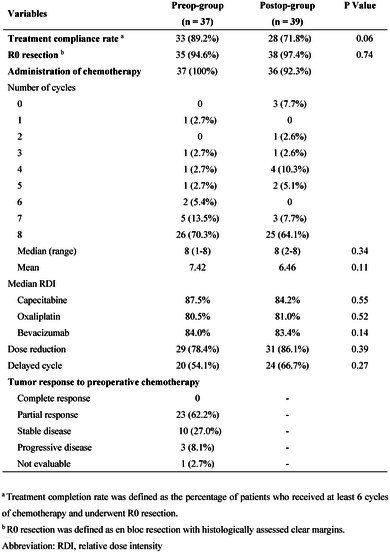

## 1 Introduction

Colorectal cancer (CRC) is a highly frequent cancer worldwide [1]. The liver is the most common site for CRC metastasis [2]. For resectable colorectal liver metastases (CRLM), liver resection is the principal curative option, significantly improving overall survival (OS). However, approximately two‐thirds of the patients relapse within the first 18 months after surgery, often with recurrence in the residual liver [3, 4].

Perioperative chemotherapy shows promise for eradicating micrometastases undetectable before surgery despite diligent imaging studies. The Fédération Francophone de Cancérologie Digestive Trial 9002, a phase III study, reported that 6 months of postoperative adjuvant chemotherapy with bolus fluorouracil (FU) plus leucovorin (LV) improved disease‐free survival in patients with resected CRLM compared with surgery alone. However, the trial was stopped prematurely because of slow patient accrual [5]. The availability of new effective chemotherapeutic agents for metastatic CRC has prompted interest in the perioperative treatment for resectable CRLM. In the European Organization for Research and Treatment of Cancer (EORTC) 40 983 Phase III [EPOC] trial, a regimen of perioperative chemotherapy (six cycles before and after the surgery) with FOLFOX4 improved the 3‐year progression‐free survival (PFS) rate compared with surgery alone [6]. According to the latest update, at a median follow‐up of 8.5 years, the median OS was longer in the chemotherapy group, although there was no significant difference [7]. Recently, the JCOG0603 trial conducted in Japan evaluated the efficacy of postoperative adjuvant chemotherapy with FOLFOX after resection of liver metastases of colorectal cancer. Regarding the primary endpoint of DFS, FOLFOX was shown to be superior to surgery alone [8]. Based on the evidence described above, NCCN and ESMO guidelines recommend up to 6 months of perioperative oxaliplatin‐based chemotherapy for patients with resectable colorectal liver metastases (CRLM) [9, 10].

Potential advantages of preoperative chemotherapy for resectable CRLM include: (1) earlier treatment of micrometastatic disease, (2) assessment of therapy responsiveness. Conversely, potential disadvantages include: (1) missing the opportunity for resection because of disease progression or achievement of a complete response, and (2) increased postoperative complications or inoperable cases due to injury to normal tissue including the liver parenchyma [11, 12].

While postoperative oxaliplatin‐based chemotherapy, the standard postoperative adjuvant for stage III CRC, has been proven to prolong OS after radical resection, it has not been shown to prolong survival after radical resection of stage IV CRLM. The integration of targeted agents into perioperative chemotherapy has been investigated as a means to improve outcomes. The addition of bevacizumab to biweekly capecitabine and oxaliplatin for six cycles in the preoperative setting achieved an objective response rate of 73.2% without increasing the surgical complication rate in a non‐randomized, single‐arm Phase II trial [13]. However, the optimal sequencing of systemic therapy and resection remains unclear. We therefore conducted a randomized phase II/III trial (The Hiroshima Surgical study group of Clinical Oncology (HiSCO)‐01); the UMIN Clinical Trial Registry (UMIN000003783) to compare the outcomes of preoperative chemotherapy (Preop‐group) with postoperative chemotherapy (Postop‐group) in patients with resectable CRLM treated with CAPOX‐Bev. Here, we report the results of the phase II part of this study.

## 2 Material and Methods

### 2.1 Endpoints

The purpose of this trial was to assess the safety, tolerability, and feasibility of the protocol treatment. The protocol treatment consisted of eight cycles of CAPOX‐Bev and R0 surgery. The primary endpoint was treatment compliance rate (TCR), defined as the proportion of patients who received six or more cycles of chemotherapy and achieved R0 resection (defined as complete resection with clear resection margins and no evidence of microscopic residual tumor). A TCR > 70% was considered acceptable. Secondary endpoints included PFS, OS, safety, as well as overall response rate (ORR) and liver damage in Preop‐group. Indocyanine green (ICG) retention rate at 15 min (ICG‐R15) was evaluated preoperatively to assess liver damage and to estimate the morbidity risk [14, 15]. PFS was defined as the time in days from randomization to the first occurrence of either disease progression or death from any cause. OS was defined as the time from randomization to death; patients alive at the last follow‐up were censored.

### 2.2 Study Design and Patients

Key inclusion criteria were age 20–80 years, histologically confirmed adenocarcinoma of the colon or rectum, no extrahepatic metastases or recurrence on computed tomography (CT), Eastern Cooperative Oncology Group performance status 0 or 1, Child‐Pugh classification A, no prior oxaliplatin‐based chemotherapy, no primary tumor‐related symptoms (e.g., bleeding or obstruction), a primary tumor that had undergone or could undergo R0 resection, sufficient organ function, and no other chemotherapy or radiotherapy, or radiofrequency ablation. Additionally, liver metastases had to satisfy at least one of the following criteria at enrollment: (1) classified as H1 or H2 (defined by the Japanese Classification of Colorectal, Appendiceal, and Anal Carcinoma, the 3rd English Edition [16])—H1 was defined as one to four metastases, all of which were 5 cm or less in maximum diameter (MD) and H2 was defined as one to four metastases, at least one of which was more than 5 cm in MD, or five or more metastases, all of which were 5 cm or less in MD, and (2) technically resectable with at least a 40% remnant liver volume after hepatectomy. Written informed consent was obtained from all patients before enrollment.

### 2.3 Procedure and Evaluation

Patients were randomly assigned (1:1; centrally by HISCO Data Center) to Preop‐group or Postop‐group by a minimization method with a random component to balance arms on the basis of institution, state of the primary tumor (resected or not resected), and the extent of CRLM (H1 or H2). Within 28 days after enrollment, protocol treatment for either the Preop‐group or Postop‐group was to be initiated. A subsequent treatment was initiated within 2–8 weeks after an initial treatment.

Patients received CAPOX‐Bev, comprising oral capecitabine 1000 mg/m^2^ twice daily from Day 1 through Day 14, an intravenous injection of oxaliplatin 130 mg/m^2^ on Day 1, and bevacizumab 7.5 mg/kg on Day 1 at 3‐weekly intervals for 8 cycles. For CAPOX‐Bev therapy, bevacizumab was prohibited in the course immediately before or after surgery due to concerns about adverse surgical effects.

A chest‐pelvis CT scan was performed every 6 weeks during the protocol treatment. In the Preop‐group, tumor response was assessed by contrast‐enhanced CT using the response criteria mentioned in the Response Evaluation Criteria in Solid Tumors (RECIST) version 1.1.

Curative‐intent surgery was mandatory. Adverse events were evaluated according to CTCAE4.0. Postoperative complications associated with liver resection were assessed according to the Clavien‐Dindo classification [17]. Posthepatectomy liver failure was defined by the International Study Group of Liver Surgery [18].

### 2.4 Statistical Analyses

The recruitment target for this study was 80 cases (40 per group) to provide 90% power under the hypothesis that treatment compliance after six cycles would have, at an expected value of 70% and a threshold value of 50%, using a one‐sided testing at a 10% significance level. TCR, ORR, and toxic effects were compared using the *χ* ^2^ test. Survival between the two treatment groups was compared using the log‐rank test, and HR using the Cox regression. In this study, an ancillary study to investigate the effect of chemotherapy on the functions of intrahepatic immune cells was analyzed separately in 30 patients and has already been reported [19].

## 3 Results

### 3.1 Patients

**FIGURE 1:**
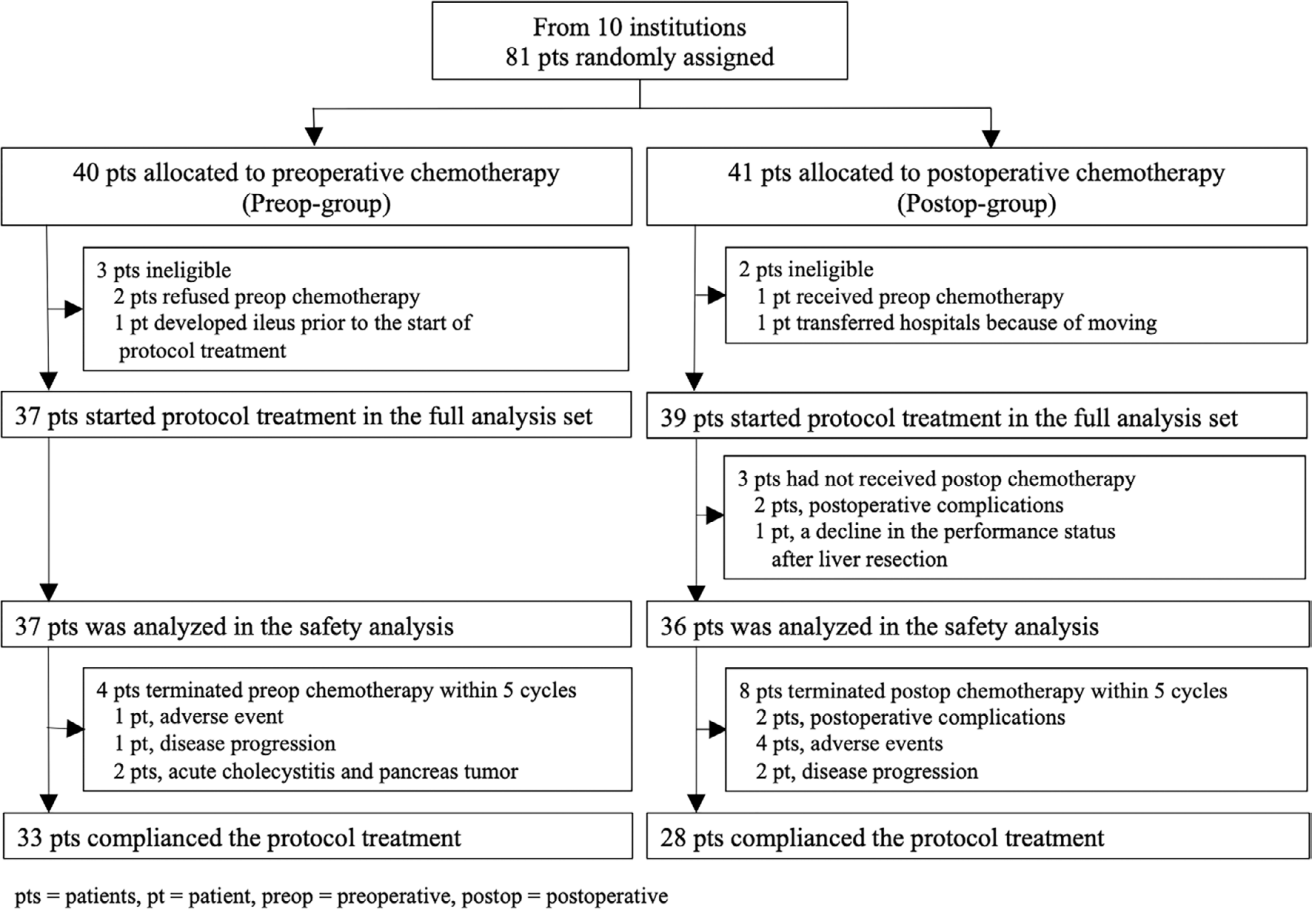
Consort flow diagram.

Between November 2010 and November 2018, 81 patients from 10 institutions were enrolled: 40 were assigned to Preop‐group and 41 to Postop‐group. Protocol treatment was not initiated in five patients (three in Preop‐group, and two in Postop‐group) after randomization, including one patient in Preop‐group who developed ileus before the protocol treatment started. Finally, 37 Preop‐group patients and 39 Postop‐group patients received the protocol treatment and were included in the full analysis set (Figure 1).

The patients' baseline characteristics are listed in Table 1. The clinical features and patient characteristics were well balanced between the two groups, except for primary tumor depth. T4 disease was present in 18.9% in the Preop‐group and 43.4% in the Postop‐group (*p* = 0.06). Primary tumors with synchronous metastases were observed in 22 patients (59.5%) in the Preop‐group and 28 patients (71.8%) in the Postop‐group. Primary tumors were resected in 20 of 22 patients (90.9%) in the Preop‐group and 26 of 28 patients (92.9%) in the Postop‐group during enrollment.

**TABLE 1:** Baseline characteristics.

Variables	Preop‐group (preoperative CTx) (*n* = 37)	Postop‐group (postoperative CTx) (*n* = 39)	*p*
Age (years)^a^	67 (44–80)	66 (27–80)	0.27
Gender			1.00
Male	26 (70.3%)	27 (69.2%)	
Female	11 (29.7%)	12 (30.8%)	
Extent of liver metastases^b^			0.80
H1	26 (70.3%)	28 (71.8%)	
H2	11 (29.7%)	11 (28.2%)	
Number of liver metastases^a^	1 (1–14)	2 (1–7)	0.75
1–4	31 (83.8%)	34 (87.2%)	
≥ 5	6 (16.2%)	5 (12.8%)	
Maximum size of tumor (cm)^a^	2.0 (0.9–7.0)	2.0 (0.8–7.7)	0.59
Synchronicity of metastases			0.47
Synchronous	22 (59.5%)	27 (69.2%)	
Metachronous	15 (40.5%)	12 (30.8%)	
T category of primary tumor			0.06
T1, T2	4 (10.8%)	2 (5.1%)	
T3	26 (70.3%)	20 (51.3%)	
T4	7 (18.9%)	17 (43.4%)	
Nodal status of primary tumor			0.64
Negative	15 (40.5%)	13 (33.3%)	
Positive	22 (59.5%)	26 (66.7%)	
Location of primary tumor			0.31
Right‐sided	8 (21.6%)	13 (33.3%)	
Left‐sided	29 (78.4%)	26 (66.7%)	
State of primary tumor			1.00
Resected	35 (94.6%)	37 (94.9%)	
Not resected	2 (5.4%)	2 (5.1%)	
Level of tumor marker at assignment			
CEA^a^	12.2 (0.7–1079)	7.2 (1.5–250)	0.23
CA19‐9^a^	19 (1–12 210)	15 (2–2144)	0.34
Adjuvant therapy after primary tumor resection			1.00
Administered	4 (10.8%)	4 (10.3%)	
Not administered	33 (89.2%)	35 (89.7%)	

^a^ Values are median (range).

^b^ H1 defined as “1–4 metastatic tumors, all of which are ≤ 5 cm in maximum diameter” and H2 defined as “1–4 metastatic tumors, at least one of which is > 5 cm in maximum diameter or five or more metastatic tumors, all of which are ≤ 5 cm in maximum diameter” according to Japanese Classification of Colorectal, Appendiceal, and Anal Carcinoma: the 3d English Edition [Secondary Publication] (14).

### 3.2 Completion and Compliance of Protocol Treatment

Treatment details are summarized in Table 2. R0 resection was performed in 38 patients (97.4%) in the Postop‐group and in 35 patients (94.6%) in the Preop‐group (*p* = 0.74). In the Preop‐group, one patient (2.7%) did not undergo surgery due to disease progression, and one patient (2.7%) underwent R1 resection. In the Postop‐group, one patient (2.6%) underwent R1 resection, and three patients did not receive postoperative chemotherapy due to postoperative complications (two patients) and a decline in performance status after liver resection (one patient) (Figure 1). A TCR of 89.2% (95% confidence interval [CI]: 74.7–96.3) and 71.8% (95% CI: 56.1–83.5) was achieved in the Preop‐group and Postop‐group, respectively (*p* = 0.06). These results satisfied the primary objective of the phase II for both groups.

**TABLE 2:** Primary endpoint and compliance, treatment tolerance, tumor response to preoperative chemotherapy.

Variables	Preop‐group (*n* = 37)	Postop‐group (*n* = 39)	*p*
Treatment compliance rate^a^	33 (89.2%)	28 (71.8%)	0.06
R0 resection^b^	35 (94.6%)	38 (97.4%)	0.74
Administration of chemotherapy	37 (100%)	36 (92.3%)	
Number of cycles			
0	0	3 (7.7%)	
1	1 (2.7%)	0	
2	0	1 (2.6%)	
3	1 (2.7%)	1 (2.6%)	
4	1 (2.7%)	4 (10.3%)	
5	1 (2.7%)	2 (5.1%)	
6	2 (5.4%)	0	
7	5 (13.5%)	3 (7.7%)	
8	26 (70.3%)	25 (64.1%)	
Median (range)	8 (1–8)	8 (2–8)	0.34
Mean	7.42	6.46	0.11
Median RDI			
Capecitabine	87.5%	84.2%	0.55
Oxaliplatin	80.5%	81.0%	0.52
Bevacizumab	84.0%	83.4%	0.14
Dose reduction	29 (78.4%)	31 (86.1%)	0.39
Delayed cycle	20 (54.1%)	24 (66.7%)	0.27
Tumor response to preoperative chemotherapy			
Complete response	0	—	
Partial response	23 (62.2%)	—	
Stable disease	10 (27.0%)	—	
Progressive disease	3 (8.1%)	—	
Not evaluable	1 (2.7%)	—	

Abbreviation: RDI, relative dose intensity.

^a^ Treatment completion rate was defined as the percentage of patients who received at least 6 cycles of chemotherapy and underwent R0 resection.

^b^ R0 resection was defined as en bloc resection with histologically assessed clear margins.

Chemotherapy was administered at a median of 1.4 (0.3–4.0) weeks from randomization in the Preop‐group and 9.3 (range 6.3–14.7) weeks from randomization in the Postop‐group. In the Postop‐group, chemotherapy started at a median of 6.2 (3.8–12.5) weeks from surgery. The mean and median numbers of cycles of CAPOX‐Bev did not significantly differ between the two groups (Preop‐group vs. Postop‐group; median 8 vs. 8, *p* = 0.34; mean 7.42 vs. 6.46, *p* = 0.11). Median relative dose intensities (RDI, minimum‐maximum) of capecitabine, oxaliplatin, and bevacizumab were 87.5% (49.4%–120.0%), 80.5% (42.1%–109.1%), and 84.0% (49.0%–124.3%) in the Preop‐group and 84.2% (37.7%–102.8%), 81.0% (12.3%–99.4%), and 83.4% (0%–100.0%) in the Postop‐group, respectively, with no statistically significant differences. Dose reduction and delayed cycles occurred in 78.4% and 54.1% of the Preop‐group and 86.1% and 66.7% of the Postop‐group, respectively.

None of the patients in the Preop‐group reported a complete response (CR). Partial response (PR) was achieved in 23 (62.2%) patients, such that the ORR was 62.2% (95% CI: 46.1–75.9). Of the 23 patients with PR, two had pathological CR. Ten patients (27.0%) had stable disease (SD) and three (8.1%) had progressive disease (PD). Of the three patients with PD, one did not undergo hepatectomy due to disease progression, but two patients underwent R0 resection. The overall disease control rate was 89.2%.

### 3.3 Feasibility of Chemotherapy

The profiles of patients who experienced Grade 3 or higher adverse events during chemotherapy are shown in Table 3. No chemotherapy‐related deaths occurred in either group. The overall incidence of adverse events was similar between the two groups (Preop‐group 16 [43.2%] vs. Postop‐group 15 [41.7%], *p* = 0.89). Hematological toxicities included thrombocytopenia in 3 (8.1%) patients in Preop‐group, with none in Postop‐group (*p* = 0.03). Non‐hematological toxicities, specifically diarrhea and nausea, were significantly more frequent in Postop‐group than in Preop‐group (diarrhea 0% vs. 8.3%, *p* = 0.04; nausea 0% vs. 8.3%, *p* = 0.04). One patient (2.8%) in Postop‐group experienced a serious adverse event associated with bevacizumab: a gastrointestinal perforation in the small bowel which was resolved with emergency surgery.

**TABLE 3:** Chemotherapy associated toxicities (≥ Grade3).

Variables	Preop‐group (*n* = 37)	Postop‐group (*n* = 36)	*p*
Any adverse events	16 (43.2%)	15 (41.7%)	0.89
Neutropenia	2 (5.4%)	5 (13.9%)	0.21
Thrombocytopenia	3 (8.1%)	0 (0.0%)	0.04
Diarrhea	0 (0.0%)	3 (8.3%)	0.04
Anorexia	1 (2.7%)	5 (13.9%)	0.07
Nausea	0 (0.0%)	3 (8.3%)	0.04
Hand‐foot syndrome	5 (13.5%)	2 (5.6%)	0.24
Peripheral neuropathy	2 (5.4%)	3 (8.3%)	0.62
Hypertension	3 (8.1%)	2 (5.6%)	0.66
Gastrointestinal perforation	0 (0.0%)	1 (2.8%)	0.23
Proteinuria	0 (0.0%)	1 (2.8%)	0.23

### 3.4 Liver Functional Reserve After Preoperative Chemotherapy

**FIGURE 2:**
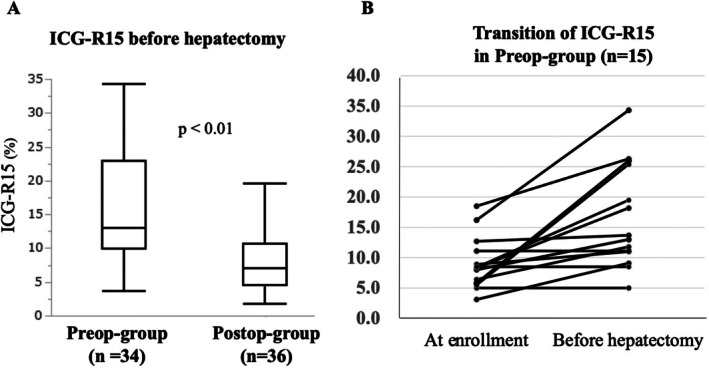
Liver functional reserve of after preoperative chemotherapy. (A) Indocyanine green retention at 15 min (ICG‐R15) before hepatectomy. (B) Transition of ICG‐R15 in Preop‐group. Preop; preoperative chemotherapy, Postop; postoperative chemotherapy.

The median ICG‐R15 before liver resection was 13.1% (range, 3.6%–42.8%) in the Preop‐group and 7.0% (range, 1.8%–19.5%) in the Postop‐group. Preoperative chemotherapy significantly increased ICG‐R15 values (*p* < 0.01; Figure 2A). We found that 12 (80.0%) of the 15 patients who underwent ICG clearance tests before and after chemotherapy had significantly increased ICG‐R15 levels (*p* < 0.01, Figure 2B and Table S1).

### 3.5 Surgery

Surgery was performed at a median of 30.2 (range, 11.8–51.6) weeks from randomization in the Preop‐group and 2.8 (range, 1.4–6.0) weeks in the Postop‐group. In the Preop‐group, surgery was performed at a median of 7.1 (range, 3.6–25.9) weeks from the last chemotherapy. R0 resection was eventually performed, although recovery from liver injury was delayed in two cases (10 and 25 weeks from last chemotherapy, respectively).

Surgical details and postoperative complications are shown in Table 4. The number and maximum size of resected liver metastases were comparable between the two groups. In the Preop‐group, the actual number of metastatic tumors resected was similar to the number of tumors before chemotherapy (Table S1). The primary tumor was simultaneously resected in two (5.6%) patients in the Preop‐group and in one (2.6%) patient in the Postop‐group (*p* = 0.52). No difference in the type of hepatectomy, duration of surgery, and estimated blood loss between the two groups was observed. No mortalities were reported in either group. Grade 2 or higher (Clavien‐Dindo classification) complications occurred in seven (19.4%) patients in the Preop‐group and in eight (20.5%) patients in the Postop‐group (*p* = 0.90). Grade 3 complications occurred in four (11.1%) patients in the Preop‐group, including intra‐abdominal abscess (two patients), anastomotic leakage of primary resection (one patient), and ileus (one patient). In the Postop‐group, grade 3 complications occurred in 3 (7.7%) patients, all of whom developed biliary fistulas. Postoperative biliary fistulas occurred only in the Postop‐group (*p* = 0.02). The occurrence of posthepatectomy liver failure was similar between the two groups.

**TABLE 4:** Surgery details and postoperative complications.

Variables	Preop‐group (*n* = 36)	Postop‐group (*n* = 39)	*p*
Number of resected liver metastases^a^	1 (1–14)	3 (1–7)	0.46
Maximum size of resected liver metastases (cm)^a^	1.2 (0–5)	1.9 (0.7–8)	0.09
Type of hepatectomy			0.40
Major	4 (11.1%)	7 (17.9%)	
Minor	32 (88.9%)	32 (82.1%)	
Simultaneous resection of primary tumor	2 (5.6%)	1 (2.6%)	0.52
Duration of surgery (min.)^a^	274 (89–687)	259 (116–623)	0.73
Estimated blood loss (gr.)^a^	196 (6–2100)	200 (10–1800)	0.90
Postoperative death	0 (0%)	0 (0%)	—
Postoperative complication			
Any Grade ≥ 2 (Clavien‐Dindo)	7 (19.4%)	8 (20.5%)	0.90
Any Grade 3 (Clavien‐Dindo)	4 (11.1%)	3 (7.7%)	0.61
Details of complications			
Biliary fistula	0 (0%)	4 (10.3%)	0.02
Post hepatectomy liver failure (ISGLS)	3 (8.3%)	3 (7.7%)	0.92
Abscess formation	3 (8.3%)	2 (5.1%)	0.57
Ascites	2 (5.6%)	0 (0%)	0.08
Ileus	2 (5.6%)	0 (0%)	0.08
Wound complication	1 (2.8%)	0 (0%)	0.30
Colonic anastomotic leakage	1 (2.8%)	0 (0%)	0.30

Abbreviation: ISGLS, International Study Group of Liver Surgery.

^a^ Values are median (range).

### 3.6 Survival

**FIGURE 3:**
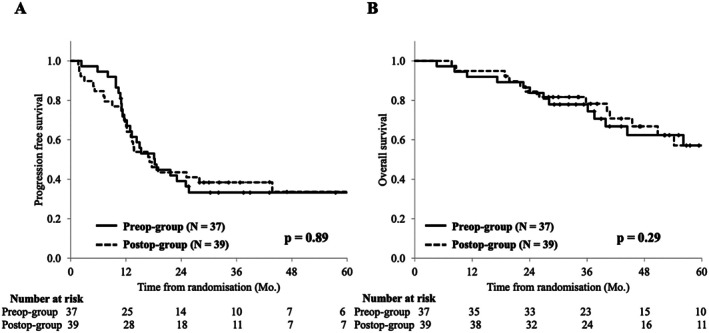
Survival curves between two groups. (A) Progression Free Survival after randomization. (B) Overall survival after randomization.

The median follow‐up period was 46 (range, 4–95) months as of July 2020. PFS analysis included 76 (67.1%) patients with 51 events: 25 (67.6%) in the Preop‐group and 26 (66.7%) in the Postop‐group. The 3‐year PFS and median PFS were 32.2% and 18.1 (95% CI: 12.2–25.7) months in the Preop‐group and 38.5% and 17.1 (95% CI: 12.1–43.7) months in the Postop‐group, with no statistically significant difference (*p* = 0.89) (Figure 3A). Twelve (32.4%) patients died in the Preop‐group and 18 (46.2%) patients in the Postop‐group. The 5‐year OS rates were 60.5% in the Preop‐group and 57.2% in the Postop‐group, with no statistically significant difference (*p* = 0.29, Figure 3B).

### 3.7 Relationship Between the Duration of Chemotherapy and Survival Outcomes

**FIGURE 4:**
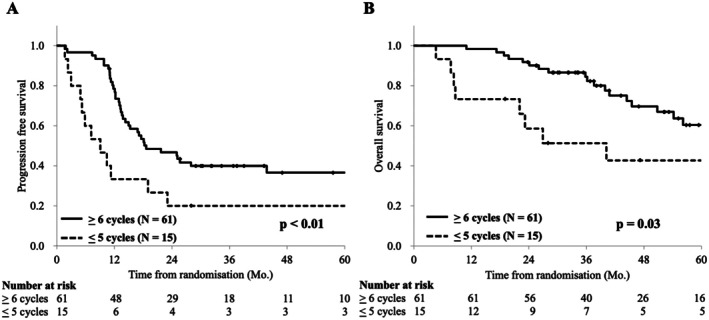
Impact of duration of chemotherapy on survival. (A) Progression free survival after randomization. (B) Overall survival after randomization. ≧ 6 cycles; six or more cycles of CAPOX‐Bev, ≦ 5 cycles five or less cycles of CAPOX‐Bev.

We conducted an ad hoc analysis to validate our hypothesis that more than 6 out of 8 cycles of chemotherapy would have a clinically meaningful effect (Figure 4). Six or more cycles of chemotherapy improved both PFS and OS as compared to five or fewer cycles in both groups. An integrated analysis showed a statistically significant prolongation of PFS (18.3 months vs. 9.0 months) and OS (78.7 months vs. 40.1 months) for patients receiving six or more cycles of chemotherapy compared to those receiving five or fewer cycles (*p* < 0.01).

## 4 Discussion

To the best of our knowledge, this is the first study to prospectively compare pre‐ and postoperative chemotherapy to assess the feasibility and safety of CAPOX‐Bev in patients with initially resectable CRLM. For resectable CRLM, preoperative chemotherapy regimens do not necessarily require a high response rate because of the difficulty in identifying the tumor during resection; rather, they may require a higher disease control rate (DCR). The ORR in the present study was 62.2%, favorably comparable to 73.2% and 66.7% reported in Gruenberger's and Nasti's studies, respectively [13, 20]. Furthermore, the overall DCR of 89.2% in the present study was also higher than that reported in the EORTC 40983 study (81%) and in the NEW EPOC study (83%), and was comparable to the 94.6% reported by Gruenberger et al. [6, 13, 21]. Moreover, this preoperative protocol achieved a high TCR of 89.2%. Preoperative chemotherapy was less toxic, except for the hand‐foot syndrome, and had a higher, though not statistically significant, RDI than postoperative chemotherapy. These results suggest that preoperative CAPOX‐Bev could be a more promising option than postoperative CAPOX‐Bev in the perioperative setting.

The primary endpoint of this study was TCR, defined as the proportion of patients who received at least six cycles of chemotherapy and achieved R0 resection. The number of courses was set at six or more because we assumed that a certain clinically meaningful efficacy could be achieved as adjuvant chemotherapy with six or more cycles, even if chemotherapy was stopped before the completion of the eighth cycle. Our ad hoc analysis showed that TCR could serve as a surrogate endpoint for feasibility (Figure 4).

Preoperative chemotherapy‐induced liver injury can impact surgical outcomes. Oxaliplatin has been linked to sinusoidal obstruction syndrome (SOS) with an incidence of up to 38% and may increase perioperative morbidity or mortality [22, 23]. Several small non‐randomized studies have reported that bevacizumab reduces SOS frequency in patients undergoing hepatectomy for CRLM, potentially lowering postoperative complication rates and enhancing operative safety [13, 20, 24, 25, 26, 27, 28]. The ICG test is useful in assessing liver dysfunction and marginal resectable liver volume [15, 29]. Our results showed that preoperative chemotherapy significantly increased the median ICG‐R15 level (13.1% vs. 7.0%, *p* < 0.01). A threshold of < 10% ICG‐R15 is recommended for major hepatectomies, such as trisectionectomy or bisectorectomy [14]. It may be best avoided if a major hepatectomy is considered for resectable CRLM in a real‐world clinical setting. The extent of liver resection must balance with the functional capacity of the remnant liver to avoid liver failure. Major hepatectomy was performed in 14.5% of patients in our study, compared to 55.5% in the EORTC 40983 study (pluri‐segmentectomy), and 36% in Gruenberger's study (three or more segments) [6, 13]. Our approach prioritized minimal resection without compromising radicality, reflecting the standard approach practiced in Japan, which emphasizes liver preservation through partial resection, unlike the Western approach where major hepatectomies such as lobectomy are primarily performed for CRLM [30, 31].

The addition of bevacizumab to the oxaliplatin‐based regimen in first‐line chemotherapy for patients with advanced or metastatic CRC improved all other efficacy outcomes, despite little improvement in response rates [24]. Therefore, bevacizumab combination could reduce the number of patients with CRLM who are inoperable due to progression of the disease with chemotherapy failure. On the other hand, bevacizumab can cause complications such as impaired wound healing, bleeding, bowel perforation, and arterial thromboembolism in the perioperative setting. In our prospective study, a few surgical complications were found to be associated with bevacizumab treatment. Biliary fistula, significantly higher in the Postop‐group, was the most common. In the Postop‐group, four patients developed biliary fistula, with two experiencing delayed bile leak during chemotherapy 3 months post‐surgery, indicating a potential association between bevacizumab and delayed healing of the bile duct, causing late dehiscence.

This study has several limitations. First, the study was terminated early without a phase III part because of insufficient patient enrollment. However, it remains valuable as a prospective study with long‐term follow‐up to confirm PFS. Second, 90.9% in the Preop‐group and 92.9% in the Postop‐group with synchronous CRLM and intact primary tumors had undergone primary tumor resection at enrollment, before receiving preoperative chemotherapy. Therefore, it remains unclear whether preoperative chemotherapy should be administered before primary tumor resection. Third, biomarker analyses for RAS, BRAF, microsatellite instability, and circulating tumor DNA were not performed. These biomarkers are crucial for determining whether to administer adjuvant chemotherapy and for selecting the optimal chemotherapy regimen.

In conclusion, our study demonstrated that preoperative CAPOX‐Bev, given in eight cycles, was compatible with R0 resection and showed a numerically higher TCR and better safety profiles than the postoperative protocol. The manageable toxicity profile of preoperative CAPOX‐Bev, along with favorable compliance, is noteworthy. This multimodal approach is highly promising for treating resectable CRLM.

## Author Contributions

**Yuji Takakura:** data curation, formal analysis, resources, software, validation, visualization, writing – original draft. **Katsunori Shinozaki:** conceptualization, investigation, methodology, project administration, supervision, writing – review and editing. **Satoshi Ikeda:** conceptualization, methodology, resources, supervision. **Hiroyuki Egi:** investigation, methodology, resources. **Yuzo Hirata:** investigation, methodology, resources. **Manabu Shimomura:** investigation, methodology, resources. **Takafumi Oshiro:** investigation, methodology, resources. **Takao Hinoi:** conceptualization, investigation, methodology, project administration, resources. **Daisuke Sumitani:** investigation, methodology, resources. **Masahiro Nakahara:** investigation, methodology, resources. **Masanori Yoshimitsu:** investigation, methodology, resources. **Naruhiko Honmyo:** data curation, software, validation, visualization. **Tsuyoshi Kobayashi:** conceptualization, funding acquisition, investigation, methodology, project administration, supervision, writing – review and editing. **Junko Tanaka:** data curation, formal analysis, software. **Hideki Ohdan:** conceptualization, funding acquisition, investigation, methodology, project administration, supervision, writing – review and editing.

## Consent

Informed consent for publication was obtained from all individual participants involved in this study.

## Conflicts of Interest

Dr. Hideki Ohdan is a current Editor or Editorial Board Member of AGSurg.

## Ethics Statement

This study was conducted in compliance with the principles of the Declaration of Helsinki and Clinical Trials Act in Japan. The study protocol and IC document were approved by the Ethical Committee for Clinical Research of Hiroshima University and the Institutional Review Board of each participating institution. All patients provided written informed consent. Informed consent was obtained from all individual participants included in this study. This trial was registered in the UMIN Clinical Trial Registry (UMIN000003783).

## Supporting information


**Table S1.** Factors before and after neoadjuvant chemotherapy in Preop‐group.
