# Supplementary material for: Preoperative Versus Postoperative Chemotherapy With CAPOX Plus Bevacizumab for Resectable Colorectal Liver Metastases: A Randomized Phase II Trial (HiSCO‐01)
Source: Ann Gastroenterol Surg. 2025 May 9;9(6):1253–62. doi: 10.1002/ags3.70035 (PMC12586932; doi:10.1002/ags3.70035)
Supplement: Supplementary file 1 — Table S1. Factors before and after neoadjuvant chemotherapy in Preop‐group. [file AGS3-9-1253-s001.doc]

**Supplementary Table S1. Factors before and after neoadjuvant chemotherapy in Preop-group**

| **Variables** | **Before chemotherapy** | **After chemotherapy** | **P Value** |
| --- | --- | --- | --- |
| Number of liver metastases ^a^ | 1 (1 - 14) | 1 (1 - 14) | 0.06 |
| Maximum size of liver metastases (cm) ^a^ | 2 (0.9 - 7) | 1.2 (0 - 5) | < 0.01 |
| Level of tumor marker |  |  |  |
| CEA (ng/ml) | 12.2 (0.7 - 1079) | 4.9 (0.8 – 104.1) | < 0.01 |
| CA19-9 (U/ml) | 19 (1 - 12210) | 16 (1 - 3586) | 0.32 |
| ICG-R15 (%) | 8.2 (3.1 – 18.5) | 13.1 (5.0 – 34.3) | < 0.01 |

Values are median (range)

^a^ The number and maximum size of liver metastases were assessed from imaging before chemotherapy and on resection specimens after chemotherapy
